# Decreasing COPD-related incidences and hospital admissions in a German health insurance population

**DOI:** 10.1038/s41598-023-48554-y

**Published:** 2023-12-02

**Authors:** Siegfried Geyer, Juliane Tetzlaff, Stefanie Sperlich, Batoul Safieddine, Jelena Epping, Sveja Eberhard, Jona Stahmeyer, Johannes Beller

**Affiliations:** 1 Medical Sociology Unit, Medizinische Hochschule Hannover, Carl-Neuberg-Strasse 1, 30625 Hannover, Germany; 2 General Local Statutory Health Insurance of Lower Saxony, Hannover, Germany

**Keywords:** Diseases, Risk factors

## Abstract

Chronic obstructive pulmonary disease (COPD) is associated with smoking and work-related health hazards. Most studies have reported prevalences, and the number of studies examining incidences and social inequalities is small. We analyzed the development of social inequalities of COPD-incidences in terms of income and exacerbations in terms of hospital admissions. Findings were based on claims data from a German statutory health insurance covering 2008 to 2019. Outpatient diagnoses were used for defining COPD-cases, hospital admissions were used for detecting exacerbations. Analyses were performed using Cox-regression. Individual incomes were depicted at three levels defined according to national averages for each year. Data of 3,040,137 insured men and women were available. From 2008 to 2019 COPD-incidences in men decreased by 42% and 47% in women. After stratification by income the reduction at the lowest income level was 41% and 50% in women. Respectively, at the highest income level reductions were 28% and 41%. Disease exacerbations decreased over time, and also social inequalities between income groups emerged. COPD-rates decreased over time at all income levels, but at a faster pace in the lowest income group, thus leading to a positive development of diminishing social gradients in men as well as in women.

## Introduction

In high-income countries incidence rates of chronic obstructive pulmonary disease (COPD) were reported to having declined over time^1^. The available evidence supports the assumption that the reduction of smoking may contribute to this development^2,3^, but for many countries evidence is unavailable. No findings have been reported for Germany, but for Spain COPD was reported to having declined between 1997 and 2007^4^. For Sweden similar developments were published for 1994 to 2012^2^, and also in Australia disease rates were declining from 1959 to 2018^5^. Exceptions from this pattern are Finland for the years 1978 to 2001^6^ and Italy for 2005 to 2009^7^, but this latter study was based on hospital data that do not permit to extend findings to the general population. Most studies on COPD were conducted without reference to particular social groups. Where social inequalities were examined, mostly cross-sectional designs had been applied^8^. Examining social differences is an obvious step since also for other smoking-related diseases social inequalities had been reported, e.g. for lung cancer as the most prominent example^9–13^. As far as evidence is available, social inequalities to the detriment of individuals from lower socioeconomic positions had been reported, and social differences were considerable. In a Canadian study the lifetime risk of receiving a COPD-diagnosis was 32% in the lowest and 23% in the highest socioeconomic group. Studies that examined the longitudinal development of social gradients reported a consistent pattern. Against the backdrop of overall diminishing disease prevalences, a study from the USA reported evidence that COPD-rates decreased in higher socioeconomic groups while they increased in the most disadvantaged social groups. Thus, social differences had widened over time^5^. So far, the available studies reported prevalences, and incidence reports are rare^14^.

Not all COPD-diagnoses are made consistently according to the same diagnostic procedures and coding practices^15^. Findings based on spirometry are considered as the best way to establish a valid diagnosis^16^, but according to a recent review paper they are also assessed on the basis of respiratory and patient-reported symptoms, expert opinions^15^ or by means of survey methods^17,18^, thus prevalence rates may vary according to the method of measurement applied^19,20^.

Against the backdrop of the considerations above we conducted a trend study to examine COPD- incidences as diagnosed based on spirometry using routinely collected health insurance data. The main topic refers to the long-term development of incidences by focusing on social disparities in terms of income. Finally, exacerbations as depicted by hospital admissions are considered.

Our overall aim was to explore trends in COPD-incidence over time. This should be achieved by pursuing four sub-objectives:

- To examine whether COPD-incidence rates are increasing or decreasing over time. Against the backdrop of decreasing tobacco consumption we expected also COPD- rates to decrease.
- To examine whether changing COPD-rates are differing by social position. Socio-economic position is depicted by income.
- To examine whether the two developments as formulated above are occurring in men as well as in women.
- Finally, to examine whether the abovementioned changes also apply to hospital admissions as indicators of exacerbated disease states.

## Data and methods

Our study is a trend study with analyses based on the complete set of claims data of a large German statutory health insurance covering all individuals aged 18 years and older insured in the calendar years 2008 to 2019. No further exclusion criteria were applied. Outpatient diagnoses were used for defining COPD-cases. AOKN insures around one third of the population in the Land of Lower Saxony. The data were drawn from the Allgemeine Ortskrankenkasse Niedersachsen (AOKN). We obtained the basis for our study as an anonymized and de-identified dataset as prepared by the AOKN.

In Germany, health insurance is mandatory for all citizens, and about 90% of the population has coverage under the regulation of the statutory health insurance system with premiums depending on income^21^. Statutory health insurances are operating under public law, and health care plans are defined uniformly according to nationwide regulations.

As the data of only one statutory health insurance were available, it had to be examined whether and to what extent the insurance population differs from the German population. This topic was examined in two studies that compared the health insurance data with national figures as issued by the German statistical office and from the Federal Employment Agency.

The first study covered the years 2004 and 2009. The proportions of men and women as well as the distribution of age did not differ from the German population. The proportion of employed individuals of men also did not differ, but in women the employment rate was lower in the insurance population. In the latter the proportion of employed in lower qualified positions was higher^22^. The second comparison covered the years 2012 and 2017. It turned out that the sex distribution did not differ between the health insurance and the German population. The proportion of individuals below 30 years of age was higher among the insured, and again a higher share of the insurance population was employed in jobs requiring lower qualification levels^23^. Nevertheless, due to the high number of individuals the insurance data covered the full range of occupations within the realm of the statutory health insurance system, i.e. individuals beyond a certain upper income limit, civil servants and officials were not represented in our data.

Routinely collected health insurance data are mainly used for accounting purposes. Thus, health-related and other behaviours as well as out-of-the pocket medications remain unrecorded as long as they are not associated with a diagnosis that is moving money. Health insurance data are left-, right or interval- censorized. Thus, beginnings or ends of observation periods may be rather arbitrary. For prevalent cases this is less important, but incidences need to be dated precisely. In order to prevent that prevalent cases are erroneously misclassified as incident ones, we counted cases only if an incident followed after an observation period of at least one year without a diagnosis.

COPD-diagnoses were coded by physicians and classified according to ICD-10: J44. As in the German health care system physicians are allowed to assign all diagnoses, we counted only cases classified as “assured” and that cases were diagnosed by internists, family physicians or pulmonologists. In order to avoid falsely assigned diagnoses, a case was counted only if a COPD- diagnosis was assigned at least twice within four quarterly periods following the first diagnosis, thus leading to underestimated COPD-rates.

The ICD-10 classification system permits to classify disease severity by coding the last two digits, but in practice the coding was not always available with sufficient accuracy. Disease exacerbations were depicted by hospital admissions^3^. We used the ICD-code and the date of admission. As for outpatient data, the episode recorded first was used for classifying cases as exacerbated.

### Identification of incident cases

Our dataset was longitudinal, thus making it possible to identify incident cases. As the data were left-, right-, and interval-censored, most cases recorded in the data are prevalent. In order to separate prevalent from incident cases and to minimize the number of cases incorrectly classified as incident, all cases from newly insured individuals were defined as prevalent. If a case was recorded following an individual year of observation without a COPD-diagnosis it was classified as incident. In the subsequent years such cases were classified as prevalent. This procedure was motivated by the attempt to identify as many correctly classified incident cases as possible. As a side-effect, some incident cases occurring in the first year of observation may be classified as prevalent, thus yielding conservative disease rates.

### Income

Income classifications were based on insurance premiums that are linearly dependent on individual income. Income was classified into categories whose cut-offs were determined according to the German average annual income of the former Western federal states of Germany in terms of the pretax salary of employed individuals as reported annually by the Federal Statistical Office of Germany (Statistisches Bundesamt). In order to make incomes of pensioners more comparable to the average German annual salary of working individuals, the average annual income was adjusted for unemployment and payments to pension funds that all employed individuals have to pay. For each year of observation, the corresponding adjusted average annual income served as a reference, and income was classified as deviation from the average annual income. Finally, individual-based income from employment as explained above was classified into three levels: 1) less than 40% of the national income, 2) 40–80% of the mean national income, and 3) more than 80% of the national income. Individuals for whom no income data were available were classified as missing. They are a heterogeneous group consisting of employed insured without income information, self-employed, family insured, pensioners, and students.

Besides income, the health insurance data are also containing education and occupation, but these are only available for employed insured. Other indicators of socio-economic status (SES), such as quality of residence, number of children or family members or working hours are unavailable.

### Statements on data use and data availability

- All methods were carried out in accordance with relevant guidelines and regulations. In the present case this applies to the STROBE guidelines.
- The data used for our study were provided by the Allgemeine Ortskrankenkasse Niedersachsen (AOKN-Local statutory Health insurance of Lower Saxony). No ethical approval by a local ethics committee was necessary. Data use was permitted according to an agreement between the first author and the AOKN. As a part of this agreement the data protection officer of the Local Statutory Health Insurance of Lower Saxony (AOKN) has approved the data use use for scientific purposes.
- No individual informed consent to use the data was required. No experiments or surveys were performed for collecting the data.

### Statistics

All analyses were performed by means of Cox proportional hazards regression^24^. This model is appropriate here as it permits it take covariates, length of observation and censoring into account. At the first step gender differences in the incidence of COPD are considered, and income as indicator of social differentiation and age are included. The main interest of our research questions referred to changes over time as depicted by the variable “year”. If multivariate models are estimated, effects of a given variable have to be interpreted as controlled for the other variables in the model. Thus, income effects have to be interpreted as estimations taking all years together. At the second step analyses are extended by stratifying by income levels, and at the final step hospitalizations as indicator of disease exacerbation are considered in separate analyses. STATA version 16.1 was used for performing the statistical analyses^25^.

### Ethics approval

Our study is based on claims data, that is, on routinely collected data. No experiments involving humans were performed. We obtained the basis for our study as an anonymized and de-identified dataset. Data preparation was performed by the AOKN. The use of this sort of data for scientific purposes is regulated by federal law, and the data protection officer of the General Local Statutory Health Insurance of Lower Saxony (AOKN) has approved its use.

## Results

The total number of subjects in the dataset was N = 3,040,137 with 1,481,485 (= 48.7%) men and 1,558,652 (= 51.3) women. The data covered all insured men and women aged 18 years and older. Tables 1 and 2 are depicting the basic figures of the variables included in the regression analyses. Annual incidence rates were below 1% and decreasing over time while the size of the insurance population was growing. Only a small proportion of patients with COPD was admitted to hospital, and these patients were considerably older when admitted to hospital than at the date of recorded onset (Tables 1 and 2). Kaplan–Meier survival estimates for incidences and for incidence-related hospital admissions and age-standardized incidence rates are documented in the appendix as additional information (see supplementary Figs. 1 and 2 and supplementary table 1).

**Table 1:** COPD-incidences for *men* with age at diagnosis, hospital admissions and income levels as indicator of socioeconomic position at an annual basis; no percentages for total numbers are given because cases are included in more than one year, depending on length of insurance periods.

		2008	2009	2010	2011	2012	2013	2014	2015	2016	2017	2018	2019
Subjects	Number	827,734	826,831	833,034	960,790	968,796	970,952	971,037	978,016	997,544	1,042,900	1,087,137	1,121,361
	Age mean/Sd, yr	49.74/ 18.21	49.77/ 18.33	49.69/ 18.42	49.45/ 18.38	49.48/ 18.46	49.54/ 18.50	48.61/ 18.52	49.60/ 18.55	49.42/ 18.56	49.01/ 18.56	48.67/ 18.52	48.53/ 18.47
COPD incidences	Number	8,094	6,910	7,839	7,465	7,091	7,247	6,603	6,455	6,151	5,329	5,227	4,967
	% *1)	0.97%	0.83%	0.93%	0.77%	0.73%	0.74%	0.68%	0.66%	0.61%	0.51%	0.48%	0.44%
	Age mean/Sd, yr	65.94/ 15.26	56.78/15.47	57.02/15.43	56.46/15.43	56.67/15.69	56.80/15.61	57.01/15.79	57.67/15.64	57.26/ 15.64	57.30/15.80	57.41/15.27	57.43/15.71
Hospital admissions	Number	146	123	84	100	126	119	91	88	90	73	72	79
	% *2)	1.80%	1.78	1.07	1.34	1.78	1.64	1.38	1.36	1.46	1.37	1.38	1.59
	Age mean/Sd, yr	70.65/10.83	71.13/ 10.93	71.16/ 10.91	70.81/ 10.83	70.47/ 11.41	70.68/ 11.42	70.51/ 11.23	70.62/ 11.21	70.14/ 11.44	70.85/ 10.89	70.62/ 10.90	70.27/ 11.19
Income levels N / %	< 40%	165,327/ 19.78%	165,736/ 19.88%	167,280/ 19.89%	187,299/ 19.34%	189,128/ 19.38%	192,559/ 19.69%	193,866/ 19.83%	193,156/ 19.62%	207,876/20.71%	217,130/20.71%	223,089/20.42	223,331/19.83%
	40–80%	239,745/ 28.68%	234,163/ 28.09%	236,142/ 28.08%	272,451/ 28.14%	277,201/28.41%	275,889/ 28.20%	276,350/ 28.27%	283,970/ 28.84%	291,507/ 29.04%	299,447/ 28.57%	306,569/28.06%	314,232/27.90%
	> 80%	222,337/ 26.60%	222,137/ 26.64%	222,021/ 26.40%	267,300/ 27.61%	272,901/ 27.96%	271,550/ 27.67%	270,731/ 27.69%	274,801/ 27.91%	281,610/ 28.06%	301,380/ 28.75%	324,463/29.70%	346,501/30.76%
	Income missing	208,419/ 24.94%	211,705/ 25.39%	215,430/ 25.62%	241,205 / 24.91%	236,657/ 24.25%	238,201/ 24.35%	236,693/ 24.21%	232,544 / 23.62%	222,702/ 22.19%	230,272/ 21.97%	238,243/21.81%	242,264/21.51%

*1) Percentages of case numbers of a given year; *2) Percentages of hospital admissions of spirometry-based incident COPD-cases of a given year yr.: years; Sd: Standard deviation.

**Table 2:** COPD-incidences for *women* with age at diagnosis, hospital admissions and income levels as indicator of socioeconomic position at an annual basis; no percentages for total numbers are given because cases are included in more than one year, depending on length of insurance periods.

		2008	2009	2010	2011	2012	2013	2014	2015	2016	2017	2018	2019
Subjects	Number	955,491	948,078	948,703	1,050,915	1,054,591	1,049,689	1,043,450	1,044,557	1,058,461	1,096,539	1,140,260	1,177,884
	Age mean/Sd, yr	54.52/ 20.29	54.49/ 20.36	54.40/ 20.42	53.84/ 20.36	53.78/ 20.41	53.78/ 20.43	53.78/ 20.43	53.71/ 20.45	53.41/ 20.44	52.92/ 20.40	52.43/ 20.32	52.11/ 20.21
COPDincidences	Number	8,622	7,279	7,983	7,286	6,852	7,205	6,325	6,350	5,903	5,254	5,164	4,884
	% *1)	0.89%	0.76%	0.83%	0.69%	0.65%	0.68%	0.60%	0.60%	0.55%	0.48%	0.45%	0.41%
	Age mean/Sd, yr	58.18/ 16.26	57.18/16.77	58.67/16.58	57.23/16.70	57.66/ 16.72	57.59/ 16.80	57.44/ 17.13	58.58/16.74	58.54/17.13	58.72/17.16	58.91/16.72	58.77/16.71
Hospital admissions	Number	107	91	82	104	84	85	83	68	67	77	54	55
	% *2)	1.24	1.25	1.03	1.43	1.23	1.18	1.31	1.07	1.14	1.47	1.05	1.13
	Age mean/Sd, yr	72.17/12.63	71.69/12.53	72.33/12.92	71.55/ 12.84	71.45/ 12.74	71.96/12.42	71.49/ 13.12	71.49/ 12.39	71.30/ 12.33	71.58/ 12.10	71.44/ 11.99	71.25/ 12.33
Income levels N / %	< 40%	349,824/ 36.28%	340,257/ 35.62%	340,564/ 35.60%	373,546 / 35.30%	376,773/ 35.50%	378,216/ 35.79%	368,708/ 35.12%	362,227/ 34.47%	390,893/36.73%	395,293/35.88%	407,067/35.54%	405,611 /34.29%
	40–80%	235,352/ 24.41%	238,669/ 24.98%	239,623/ 27.05%	239,623 / 24.55%	262,794/24.76%	263,201/ 24.90%	267,886/ 25.52%	281,483/ 26.78%	288,468/ 27.10%	303,461/ 27.54%	317,559/27.72%	337,615/28.54%
	> 80%	68,284 / 7.08%	73,775/ 7.72%	75,242/ 7.86%	75,242 / 7.79%	84,988/ 8.01%	85,660/ 8.10%	88,932/ 8.47%	93,420/ 8.89%	98,889/ 9.29%	111,667/ 10.14%	124,447/10.86%	139,412/11.79%
	Income missing	310,653/ 32.22%	301,257/ 31.68%	215,430/ 31.49%	301,257 / 32.36%	336,888/ 31.74%	329,817/ 31.21%	324,249/ 30.89%	313,777/ 29.86%	286,114/ 26.88%	291,372/ 26.45%	296,351/25.87%	300,130/25.38%

*1) Percentages of case numbers of a given year; *2) Percentages of hospital admissions of spirometry-based COPD-cases of a given year; yr.: years; Sd: Standard deviation.

## Regression analyses

### Analyses with income, year of observation, and age (Table 3).

**Table 3:** COPD-incidences by year of observation and income in men and women: Hazard ratios (HR), standard errors and confidence intervals.

Year	Men			Women		
	Hazard ratio	*p*	95% CI	Hazard ratio	*p*	95% CI
2008	1	–	–	1	–	–
2009	0.9	< 0.001	0.86–0.94	0.9	< 0.001	0.86–0.94
2010	1.05	0.04	1.00–1.10	1.03	0.28	0.98–1.08
2011	0.87	< 0.001	0.83–0.90	0.9	< 0.001	0.86–0.93
2012	0.84	< 0.001	0.81–0.88	0.83	< 0.001	0.79–0.86
2013	0.9	< 0.001	0.86–0.94	0.9	< 0.001	0.86–0.95
2014	0.82	< 0.001	0.78–0.85	0.81	< 0.001	0.78–0.85
2015	0.82	< 0.001	0.77–0.85	0.81	< 0.001	0.78–0.86
2016	0.72	< 0.001	0.69–0.76	0.7	< 0.001	0.67–0.74
2017	0.64	< 0.001	0.61–0.67	0.64	< 0.001	0.61–0.68
2018	0.62	< 0.001	0.59–0.65	0.59	< 0.001	0.56–0.62
2019	0.58	< 0.001	0.55–0.60	0.53	< 0.001	0.51–0.56
Income < 40%	1	–	–	1	–	–
level 40–80%	0.73	< 0.001	0.72–0.74	0.93	< 0.001	0.92–0.95
> 80%	0.67	< 0.001	0.66–0.68	0.84	< 0.001	0.82–0.86
Missing	0.78	< 0.001	0.76–0.79	0.85	< 0.001	0.84–0.87
Age (months)	1.002	< 0.001	1.0019–1.0020	1.0011	< 0.001	1.0010–1.0011

At the first step regression analyses with gender and age as covariates were performed in order to decide whether it was appropriate to differentiate between men and women. after controlling for income, a hazard ratio (HR) of HR = 0.81 (p < 0.001; 95%-confidence interval: 0.80–0.82) emerged, i.e. men were more likely to obtain a COPD-diagnosis than women. The numeric HR also remained unchanged after having extended the model and after having included year of diagnosis as independent variable. No separate table is displayed for these findings.

In men COPD- rates were decreasing over time, although a monotonous decrease emerged only in the years 2013 and later. Finally, compared with 2008 as reference year, the rates of 2019 dropped to a level of 58%. A social gradient in terms of income emerged as the rate of the intermediate and the highest income category decreased to 73% and 63% compared with the lowest category. In women the rates were also decreasing over time. A social gradient in terms of income emerged also in women. The gradient was however somewhat smaller than in men.

At the second step social disparities for obtaining a COPD-diagnosis were considered after stratification by income. Table 3 revealed that the hazard ratios for obtaining a diagnosis decreased with increasing income level, i.e., over the whole observation period a significant social gradient emerged. With respect to time, the HRs were indicating decreasing risks for a COPD-diagnosis as time comes closer to the present, although monotonously decreasing HRs emerged only after 2013, and this held for men as well as for women. In men as well as in women the hazard ratio for the year 2010 was higher than in the preceding and in the following year.

The proportional hazards-assumption was tested based on Schoenfeld residuals. At first the whole model as depicted in Table 3 was considered, and it turned out that, with the exception of the year 2019, the tests for years of observation were not statistically significant. After Bonferroni-correction the overall test also turned out as statistically insignificant. If the analyses were confined to year of observation, the proportional hazards-assumption was fulfilled, and the effects for year of observation were stronger. For income, the results were mixed. For the male and for the female study population the tests were insignificant in the multivariate model after Bonferroni-correction, if income was tested alone, the proportional hazards- assumption was not fulfilled. For age the assumption was not met either.

### The development of COPD-rates over time stratified by income

At the third step stratified analyses were performed for each income level in order to examine whether rates are decreasing at all levels (Table 4).

**Table 4:** COPD-incidences by year of observation and income in women and men.

Year 2008	Men			Women		
	HR	*p*	95% CI	HR	*p*	95% CI
	Ref	–	–	1	–	–
**Income level < 40% of the national average**						
2009	0.86	0.001	0.79–0.94	0.85	< 0.001	0.78–0.92
2010	1.05	0.32	0.96–1.14	0.95	0.22	0.88–1.03
2011	0.86	< 0.001	0.80–0.94	0.82	< 0.001	0.76–0.88
2012	0.77	< 0.001	0.71–0.84	0.76	< 0.001	0.71–0.82
2013	0.88	0.001	0.81–0.96	0.84	< 0.001	0.78–0.91
2014	0.83	< 0.001	0.77–0.92	0.75	< 0.001	0.69–0.81
2015	0.83	< 0.001	0.76–0.91	0.75	< 0.001	0.69–0.81
2016	0.72	< 0.001	0.66–0.78	0.66	< 0.001	0.61–0.72
2017	0.65	< 0.001	0.59–0.71	0.61	< 0.001	0.56–0.66
2018	0.64	< 0.001	0.59–0.71	0.56	< 0.001	0.51–0.60
2019	0.59	< 0.001	0.54–0.65	0.5	< 0.001	0.46–0.54
Age (months)	1.021	< 0.001	1.0206–1.0220	1.0083	< 0.001	1.0078–1.0088
**Income level 40–80% of the national average**						
Year 2008	1	–	–	1	–	–
2009	0.89	0.02	0.81–0.98	0.82	< 0.001	0.74–0.92
2010	1.02	0.75	0.92–1.11	1.19	0.002	1.07–1.32
2011	0.81	< 0.001	0.75–0.88	0.88	0.02	0.81–0.98
2012	0.83	< 0.001	0.76–0.91	0.88	0.02	0.80–0.98
2013	0.84	< 0.001	0.76–0.92	0.9	0.05	0.81–0.99
2014	0.77	< 0.001	0.70–0.84	0.92	0.1	0.83–1.02
2015	0.75	< 0.001	0.69–0.83	0.75	0.03	0.81–0.99
2016	0.67	< 0.001	0.61–0.73	0.89	< 0.001	0.71–0.88
2017	0.62	< 0.001	0.56–0.68	0.82	< 0.001	0.74–0.91
2018	0.58	< 0.001	0.54–0.64	0.7	< 0.001	0.63–0.78
2019	0.54	< 0.001	0.49–0.59	0.62	< 0.001	0.56–0.69
Age (months)	1.0206	< 0.001	1.0200–1.0212	1.0139	< 0.001	1.0134–1.0145
**Income level > 80% of the national average**						
2009	0.97	0.55	0.87–1.08	0.95	0.58	0.79–1.14
2010	1.18	0.01	1.01–1.32	1	0.99	0.83–1.20
2011	0.94	0.16	0.86–1.03	0.98	0.78	0.82–1.16
2012	0.92	0.08	0.83–1.01	0.87	0.11	0.73–1.03
2013	1.08	0.13	0.98–1.20	1	0.99	0.84–1.20
2014	0.91	0.06	0.83–1.01	0.75	0.01	0.62–0.90
2015	0.99	0.91	0.90–1.10	0.87	0.13	0.73–1.04
2016	0.88	0.02	0.80–0.98	0.78	0.001	0.66–0.94
2017	0.81	< 0.001	0.73–0.90	0.69	< 0.001	0.58–0.83
2018	0.75	< 0.001	0.68–0.82	0.61	< 0.001	0.52–0.72
2019	0.72	< 0.001	0.65–0.79	0.59	< 0.001	0.51–0.69
Age (months)	1.0298	< 0.001	1.0289–1.0307	1.0202	< 0.001	1.0190–1.0214
**Income data missing**						
2009	0.93	0.12	0.85–1.02	1	0.91	0.93–1.09
2010	1.03	0.42	0.95–1.13	1.02	0.56	0.95–1.11
2011	0.89	0.01	0.82–0.96	0.96	0.28	0.90–1.03
2012	0.89	0.01	0.82–0.97	0.84	< 0.001	0.78–0.90
2013	0.89	0.01	0.81–0.96	0.95	0.22	0.88–1.03
2014	0.8	< 0.001	0.73–0.87	0.82	< 0.001	0.76–0.89
2015	0.76	< 0.001	0.69–0.83	0.83	< 0.001	0.76–0.89
2016	0.68	< 0.001	0.62–0.74	0.7	< 0.001	0.62–0.73
2017	0.55	< 0.001	0.50–0.60	0.55	< 0.001	0.50–0.60
2018	0.54	< 0.001	0.49–0.60	0.53	< 0.001	0.49–0.58
2019	0.49	< 0.001	0.45–0.54	0.48	< 0.001	0.44–0.52
Age (months)	1.032	< 0.001	1.0312–1.0328	1.021	< 0.001	1.0202–1.0217

In men the rates of the lowest income group were decreasing from 2008 to 2019, and finally the 2019-rate dropped down to 59% as compared to 2008 as reference year, although the development before 2013 was not steady. The same held for the intermediate income group with incomes between 40 and 80% of the national level. This holds only partly for the highest income group, where rates also dropped, but the rates changed inconsistently, and only for the last four years they were statistically significant. In the group of men where no income information was available the rates also dropped over time.

In women a consistent decrease of COPD-rates emerged in the lowest income group, and in 2019 the COPD-rate dropped down to 50% of the rate in 2008. The same development occurred in the intermediate and in the highest income group reaching 62% and 59% of the 2008-rate. As reported for men, the rates in the high-income group developed inconsistently, and the differences to 2008 became statistically significant only after 2015. The development of the group without income data were also following a downward trend.

As already mentioned in the first regression analyses, the HRs for 2010 were higher than in the preceding and in the following year. In the stratified analyses this was found again. In men this applied to the highest, in women in the intermediate income group.

If the long-term development of hazard ratios by income in men are considered, the developments of the lowest and the intermediate income groups were developing in a similar way. The figures were developing downwardly, and the development of unclassified subjects was also pointing towards the same direction. The exception was the highest income group that started with the lowest proportions of COPD, and in this group the smallest decrease occurred. While the lowest income group ended with 53% of the reference year, the respective figure in the intermediate income group was 49%. The lowest decrease occurred in the highest income category with 70% of the reference year. However, it has to be borne in mind that in the latter group the proportion of individuals with COPD was lowest.

The proportional hazards-assumption was tested for every regression analysis depicted in Table 4. In no case the test turned out to be significant for year of observation. With the exception of the intermediate income group in women, the tests for age were again statistically significant.

### Hospitalizations

As displayed in Tables 1 and 2, for newly diagnosed COPD-patients the number of inpatient cases had decreased over time. If analyzed by means of regression analyses, hospitalization risks of men and of women were decreasing over the observation period 2008 to 2019, reaching 47% of the rate in 2019 (Table 5). In a separate analysis without stratification (details not shown in a table) rates decreased continuously, but there was a marked gender difference as age-controlled rates in women were 47% lower than in men (HR = 0.53; p < 0.001; 95%CI = 0.52–0.54). Apart from decreasing rates over time, also social differences in terms of income emerged, and this held in women as well as in men. In the latter the incidence rate in the lowest income group was 76% lower than in the group with the highest income. In women income differences were smaller, but nevertheless statistically significant.

**Table 5:** Hospital admissions due to a main diagnosis of COPD by year of observation and income level in men and women: Hazard ratios, standard errors and confidence intervals.

Year	Men			Women		
	Hazard ratio	p	95% CI	Hazard ratio	p	95% CI
**2008**	1	–	–	1	–	–
2009	0.97	0.67	0.84–1.12	0.95	0.6	0.80–1.14
2010	0.87	0.04	0.74–1.00	0.89	0.23	0.74–1.07
2011	0.76	0.06	0.67–0.85	0.88	0.1	0.76–1.03
2012	0.75	< 0.001	0.66–0.86	0.76	0.001	0.64–0.89
2013	0.78	< 0.001	0.68–0.90	0.78	0.001	0.66–0.92
2014	0.64	< 0.001	0.56–0.73	0.71	< 0.001	0.60–0.83
2015	0.57	< 0.001	0.49–0.65	0.76	0.001	0.64–0.90
2016	0.58	< 0.001	0.50–0.67	0.59	< 0.001	0.49–0.70
2017	0.48	< 0.001	0.41–0.56	0.66	< 0.001	0.55–0.79
2018	0.48	< 0.001	0.41–0.56	0.5	< 0.001	0.41–0.60
2019	0.47	< 0.001	0.41–0.55	0.53	< 0.001	0.44–0.64
**Income** < 40%	1	–	–	1	–	–
**level** 40–80%	0.62	< 0.001	0.60–0.64	0.87	< 0.001	0.84–0.90
> 80%	0.24	< 0.001	0.23–0.26	0.53	< 0.001	0.49–0.58
Missing	0.52	< 0.001	0.50–0.55	0.74	< 0.001	0.70–0.78
Age (months)	1.005	< 0.001	1.0050–1.0051	1.0037	< 0.001	1.0037–1.0039

The proportional hazards-assumption was again fulfilled for year of observation, and this also holds for all income strata after Bonferroni-correction for men as well as for women. For age it was not fulfilled.

## Discussion

Our study focused on time trends and social inequalities in the incidence of COPD. It broadens existing knowledge in several ways: No studies on incidences have been published so far as earlier work was focused on prevalence rates, and ours is also the first one study to use data from Germany. The findings were based on a longitudinal dataset from a large statutory health insurance, thus making it possible to analyze changes occurring over a longer time period. In contrast to survey-based studies our data were covering a complete population where health-related nonresponse does not occur. In addition, large case numbers are leading to smaller confidence intervals, and they are reducing the risk of falsely overestimating outliers.

We found that incidence rates were decreasing over time, women had lower rates than men, and COPD-rates differed by income level. Smoking rates in Germany decreased in men and in women, but only in men tobacco-associated cancer rates were reported to having decreased significantly^26,27^. It needs to be discussed how changing rates in our data can be interpreted. Over the 11 years covered the COPD-incidence in men dropped by 42% and 47% in women. These magnitudes are within the ranges reported in earlier studies. For Spain the prevalence rates of moderate to severe COPD (according to GOLD-standards) were reported to having decreased by 50% over a decade^4^, and for Sweden a reduction of 30% prevalent cases were reported for 1994 to 2009^2^. Comparing these studies with our findings yields only an incomplete picture because they reported prevalence rates while our study was based on incidences. A methodologically sound Swedish study examined incidence rates over an observation period of 10 years. It started with subjects with non-serious respiratory symptoms and compared three birth-cohorts (1919/1920, 1934/1935 and 1949/1950). After a questionnaire-based screening COPD was confirmed by spirometry. The initial screening made it possible to record also risk factors and preclinical symptoms. Finally, depending on the criteria applied, cumulative 10-year incidences of 8.2% and 13.5% were reported^14^.

In the results-section it was reported that the HRs for 2010 did not fit into the general downward development of incidence rates. Although a concluding explanation is difficult, we assume that this may be due to reporting routines within the health insurance, i.e., in a large number of cases delayed reporting might have occurred, thus cases appeared only in the following year.

Our data do not include health-related behaviours and symptoms that are not presented to a physician, but they permitted to study the development of hospitalizations as indicators of exacerbations. The proportion of subjects admitted to hospital finally turned out to be small.

COPD-rates also turned out to increase with decreasing income level. So far, our findings on social inequalities in COPD are in line with findings pertaining to other smoking-related diseases with the most prominent example being lung cancer^9,12,28,29^. Separate analyses by income level revealed that in the highest income group no or only minor decreases in individual rates occurred. Significant changes were found only in the intermediate and in the lowest income group. In sum, this development led to a decrease of social gradients mainly in men that was driven by the intermediate and the lowest income group. A report from the USA came to different conclusions. The lowest socioeconomic groups were also driving changing social gradients, but in contrast to our findings the disease rates in the lowest socio-economic group had increased, thus resulting in widening income- related health disparities^5^. The study setting was however different from ours since COPD had not been diagnosed by spirometry, and also other respiratory diseases and symptoms had been recorded. In the literature decreasing COPD-rates were explained by smoking as the most important risk factor that also has a social gradient. In Germany the consumption of nicotine has decreased in the last decades as between 1991 and 2016 the sale of cigarettes had dropped by 30%. Also among smokers the proportion of heavy consumers was reduced by about 50% from 1998 to 2014^30^. Between 1999 and 2013 the reduction of tobacco consumption in Germany was mainly driven by developments in the population holding middle and high socioeconomic positions^31^. Regardless of a reduction of nicotine consumption, individuals in lower socioeconomic positions might also be particularly affected by occupation-related exposures such as dust, chemicals, vapours and fumes^32^, but there is evidence that significant improvements following workplace interventions in Europe have taken place^33^. Reductions of work-related health hazards are mainly directed towards improving conditions of men holding lower occupational positions what may explain the favourable developments in lower income groups.

In our study we used only income as indicator of socio-economic status^34^, because it had the lowest number of missing values. In health insurance data information on education and occupation are available only for employed insured, and considering these indicators would have led to higher numbers of missings. Thus, we considered only one aspect of social differentiation, although education and occupation as two more prominent and frequently used SES-indicators are depicting different aspects of socio-economic position^35,36^. An alternative approach might be to use classifications of occupations. This approach does however not permit to characterize workplaces with respect to related hazards with sufficient precision, and this type of classification must also take long-term changes of expositions into account. It might be discussed whether the application of age, period and cohort models are a useful extension of our analytic strategy^37^. We finally decided against it because our interest was directed towards comparing the development of COPD only over different time periods at population level. Using age, period and cohort models would also have given rise to the often discussed issue whether period and cohort effects can be separated^38^.

Our study has some potentially limiting conditions that have to be discussed. Our incidence rates are underestimated because diagnoses in the first year of observation remained undetected because falsely identified incident cases should be excluded as far as possible, thus leading to conservative incidence rates. A second source of underestimation may be that COPD-cases had only been diagnosed on the basis of spirometry without also having considered patient-reported symptoms as indicators. The decision not to use symptoms was based on the last update on the GOLD-report from 2022^39^. According to these guidelines, also patient-reported symptoms may be used for making a COPD-diagnosis, but the final decision must be backed by spirometry. Apart from hospital admissions, disease severity could not be determined because the recorded ICD-10-diagnoses were lacking sufficient precision. Unfortunately, claims data do not include behavioural measures, and this refers to the abovementioned risk factors, e.g. smoking or the presence of workplace hazards such as dust, gas, fumes, chemical agents and urban air pollution that can only be drawn from other types of data^3,40^. Although for Europe directives on safety and health at work (https://osha.europa.eu/en/european-standards) have been issued, data are either unavailable or published only at cross-sectional level^41^. Nevertheless, we can conclude that social gradients in terms of COPD-incidences were decreasing over time, and this development was mainly due to the middle and income groups what makes the development of Germany different from rate changes reported for North America. Taken together, our findings are indicating a positive change in the area of health inequalities, and analyses of data pertaining to the following years will reveal whether this development will continue.

### Supplementary Information

Supplementary Information 1.

Supplementary Information 2.

Supplementary Information 3.

## Data Availability

The datasets generated and analysed during the current study must not be made publicly available due to laws governing the protection of privacy of the insured individuals by the Statutory Local Health Insurance of Lower Saxony (AOKN). Interested researchers can send data access requests to Jona Stahmeyer at the AOKN as the responsible officer for data affairs. He can be reached via the following e-mail address: jona.Stahmeyer@aok.nds.de.
