# Supplementary material for: Decreasing COPD-related incidences and hospital admissions in a German health insurance population
Source: Sci Rep. 2023 Dec 2;13:21293. doi: 10.1038/s41598-023-48554-y (PMC10693544; doi:10.1038/s41598-023-48554-y)
Supplement: Supplementary file 1 — Supplementary Information 1. [file 41598_2023_48554_MOESM1_ESM.docx]

**Supplementary figure 1: Kaplan-Meier survival estimates for COPD-incidences; analysis time counted in days**
