# Supplementary material for: Decreasing COPD-related incidences and hospital admissions in a German health insurance population
Source: Sci Rep. 2023 Dec 2;13:21293. doi: 10.1038/s41598-023-48554-y (PMC10693544; doi:10.1038/s41598-023-48554-y)
Supplement: Supplementary file 3 — Supplementary Information 3. [file 41598_2023_48554_MOESM3_ESM.docx]

**Supplementary table 1: Frequencies and age-standardized incidence rates 1000 individuals for men and women for 2008 to 2019**

|  | **Men, frequencies and incidence rates per 1000 individuals** | | | | | | | | | | | |
| --- | --- | --- | --- | --- | --- | --- | --- | --- | --- | --- | --- | --- |
| Age | 2008 | 2009 | 2010 | 2011 | 2012 | 2013 | 2014 | 2015 | 2016 | 2017 | 2018 | 2019 |
| 18-27 years | 379/  3.00 | 363/  2.80 | 396/  3.00 | 384/  2.50 | 377/  2.40 | 361/  2.30 | 350/  2.30 | 305/  0.20 | 327/  2.10 | 256/  1.60 | 227/  1.30 | 334/  1.80 |
| 28-37  years | 499/  4.44 | 451/  4.1 | 501/  4.40 | 512/  3.70 | 501/  3.50 | 528/  3.60 | 482/  3.20 | 443/  2.90 | 415/  2.50 | 416/  2.30 | 373/  1.90 | 456/  2.10 |
| 38-47  years | 1269/  7.61 | 1091/ 6.70 | 1157/  7.3 | 1134/ 6.30 | 1043/ 6.00 | 1035/ 6.20 | 892/  5.60 | 782/  5.00 | 731/  4.70 | 645/  4.10 | 608/  3.70 | 703/  3.80 |
| 48-57  years | 1772/  12.00 | 1620/ 10.70 | 1917/ 12.30 | 1818/ 10.00 | 1709/  9.1 | 1780/  9.30 | 1626/  8.40 | 1639/  8.30 | 1560/  10.20 | 1337/  6.40 | 1345/  6.30 | 1657/  6.50 |
| 58-67  years | 1796/ 16.01 | 1436/ 12.90 | 1639/ 14.50 | 1632/ 12.60 | 1513/ 11.50 | 1660/ 12.10 | 1492/  10.60 | 1524/  10.50 | 1508/  10.20 | 1298/  8.40 | 1359/  8.40 | 1714/  7.50 |
| 68-77  years | 1814/  15.40 | 1465/ 12.40 | 1630/ 14.00 | 1430/ 10.90 | 1383/ 10.9 | 1255/  10.40 | 1196/  10.40 | 1109/  9.90 | 1029/  9.60 | 850/  8.00 | 803/  7.60 | 1020/  6.30 |
| 78+  years | 638/ 10.90 | 548/  9.00 | 647/ 10.40 | 6180/ 0.89 | 612/  8.30 | 673/  8.70 | 628/  7.80 | 702/  8.30 | 618/  7.00 | 576/  6.20 | 549/  5.70 | 832/  5.30 |
|  | **Women, frequencies and incidence rates per 1000 individuals** | | | | | | | | | | | |
| Age | 2008 | 2009 | 2010 | 2011 | 2012 | 2013 | 2014 | 2015 | 2016 | 2017 | 2018 | 2019 |
| 18-27  years | 455/  3.80 | 441/  3.60 | 414/  3.30 | 419/  3.00 | 369/  2.60 | 421/  2.90 | 398/  2.80 | 316/  2.20 | 323/  2.20 | 293/  1.90 | 264/  1.70 | 362/  2.10 |
| 28-37  years | 585/  5.30 | 542/  5.00 | 474/  4.30 | 522/  4.10 | 507/  3.90 | 519/  9.30 | 473/  3.50 | 426/  3.10 | 434/  2.90 | 385/  2.40 | 348/  2.00 | 489/  2.40 |
| 38-47  years | 1196/  7.60 | 1138/  7.40 | 1144/  7.60 | 1149/  7.00 | 993/  6.20 | 1019/  6.60 | 892/  6.00 | 859/  6.00 | 717/  5.00 | 608/  4.10 | 607/  3.90 | 829/  4.60 |
| 48-57  years | 1756/  12.10 | 1497/  10.20 | 1655/  11.00 | 1591/  9.30 | 1508/  8.60 | 1658/  9.30 | 1436/  7.90 | 1400/  7.60 | 1342/  7.20 | 1185/  6.10 | 1178/  5.80 | 1810/  6.90 |
| 58-67  years | 1672/  13.80 | 1294/  11.00 | 1563/  13.20 | 1330/  10.00 | 1302/  9.60 | 1460/  10.40 | 1230/  8.60 | 1330/  9.10 | 1186/  7.90 | 1168/  7.50 | 1199/  7.30 | 1834/  7.50 |
| 68-77  years | 2038/  12.5 | 1576/  9.80 | 1745/  11.10 | 1494/  8.90 | 1334/  8.30 | 1286/  8.50 | 1069/  7.50 | 1128/  8.30 | 997/  7.70 | 786/  6.20 | 781/  6.30 | 1099/  5.80 |
| 78+  years | 952/  6.30 | 825/  5.50 | 1062/  7.10 | 830/  5.20 | 860/  5.30 | 922/  5.60 | 857/  5.10 | 929/  5.50 | 972/ 5.60 | 874/  5.00 | 852/  4.80 | 1313/  5.00 |
